# StepsConnect: A Real-Time Step-Sensing Ambient Display System to Support Connectedness for Family Members Living Apart

**DOI:** 10.3390/s26051726

**Published:** 2026-03-09

**Authors:** Rui Wang, Tianqin Lu, Feng Wang, Yuan Lu, Jun Hu

**Affiliations:** 1 School of Design, Jiangnan University, Wuxi 214122, China; 2 Department of Industrial Design, Eindhoven University of Technology, 5600 MB Eindhoven, The Netherlands

**Keywords:** ambient awareness system, informative artwork, remote family connectedness

## Abstract

Physical separation between family members arises not only from life choices such as education and employment, but also from health-related constraints that limit physical co-presence. This paper presents StepsConnect, a real-time step-sensing-based ambient display system that transforms personal walking data into dynamic digital art, providing low-effort and non-intrusive presence cues for family members living apart. The system continuously captures step data via smartphones and renders them as spatial and embodied visual cues embedded in everyday environments. We conducted a 90 min laboratory study with 15 young adult–parent dyads, in which young adults engaged in a simulated work session while viewing real-time visualizations of their parents’ step activity. Young adults’ perceived connectedness was measured using the Inclusion of Other in the Self (IOS) scale and complemented with semi-structured interviews, while parents’ walking data were logged to provide an objective behavioral reference. Quantitative results indicated modest and heterogeneous changes in IOS scores at the group level, with individual variability across participants. Qualitative findings suggested that step-based visualizations primarily functioned as ambient reminders and cues of presence, supporting momentary relational awareness while remaining calm and non-intrusive within the workspace context. Walking data exhibited large variation across dyads, providing objective context for participants’ subjective experience of presence, although connectedness was not simply proportional to activity magnitude. The findings suggest that aesthetic step-based ambient visualization primarily supports momentary relational awareness rather than immediate shifts in stable closeness. By clarifying this distinction, the study advances understanding of how sensing-based digital art may function as a complementary presence layer in intergenerational contexts.

## 1. Introduction

Nowadays, many young adults experience geographical separation from their parents due to education, employment, or other life transitions [1]. Such separation can gradually influence feelings of belonging and connectedness within families [2,3,4]. Because social connectedness plays an important role in psychological and physical wellbeing [5,6,7,8,9], sustaining relational bonds across distance has become an important design challenge.

Digital communication technologies, including video calls, messaging applications, and social media, are widely used to maintain contact between family members living apart [10]. However, these tools typically require intentional effort, synchronized availability, and emotional energy [11,12]. Prior research has shown that mismatched expectations regarding communication frequency may lead to frustration or communication fatigue [13,14,15,16]. Moreover, conventional tools primarily support deliberate exchanges, offering limited means to share subtle, ongoing cues of everyday activity that contribute to a sense of presence and companionship awareness [10,17,18]. Supporting low-effort, continuous relational awareness therefore remains an open challenge.

Ambient and low-effort communication systems provide one potential direction. Prior work has demonstrated that activity-based cues, such as movement, light patterns, or shared environmental signals, can support peripheral awareness of others’ daily lives without requiring direct interaction [19,20,21,22,23]. Such systems aim to cultivate a sense of “co-presence,” in which individuals perceive others as simultaneously active within a shared or mediated space [22,24,25,26].

Human perception of social presence is strongly influenced by bodily movement and spatial cues [27,28]. Embodied simulation theory suggests that observing another person’s movement can activate sensorimotor representations associated with action understanding and social perception [29,30,31,32,33]. Experimental and neurophysiological studies consistently report measurable differences between biologically plausible human motion and non-biological control stimuli, with medium-to-large behavioral effects and robust sensorimotor activation patterns [34,35,36,37,38,39,40,41,42,43]. Although these findings operate at neural and perceptual levels distinct from the present design context, they collectively suggest that bodily movement cues are perceptually salient and socially meaningful.

In mediated environments, providing embodied and spatially organized activity cues may therefore support relational awareness across distance [36,44,45,46,47,48]. Systems that visualize everyday bodily activity have shown that perceiving another person as “active” can function as a lightweight presence signal while avoiding the burden associated with constant messaging [19,47,49,50,51]. Walking, in particular, represents a naturally occurring and continuously generated bodily activity that reflects ongoing participation in everyday life. When rendered within a shared visual display, step-based information may operate as an embodied cue of liveness and presence.

Beyond the data itself, the manner in which activity information is visually represented plays a critical role in shaping interpretation and emotional engagement. Prior work has visualized activity through numerical indicators [50], textual updates [47], simple graphical displays [51], or embedded signals in everyday objects (e.g., lamps [26], cushions [25], canes [21]). These approaches demonstrate different strategies for embedding activity information into domestic environments. However, relatively limited empirical work has examined how digital art-based visualization functions as a representational layer for bodily activity cues.

Digital art offers an alternative representational strategy. Rather than presenting activity data in numerical or diagrammatic form, artistic visualization can translate bodily signals into dynamic, metaphorical, and aesthetically mediated expressions. Such representations may influence how activity cues are interpreted, imagined, and emotionally contextualized within everyday environments.

**Figure 1:**
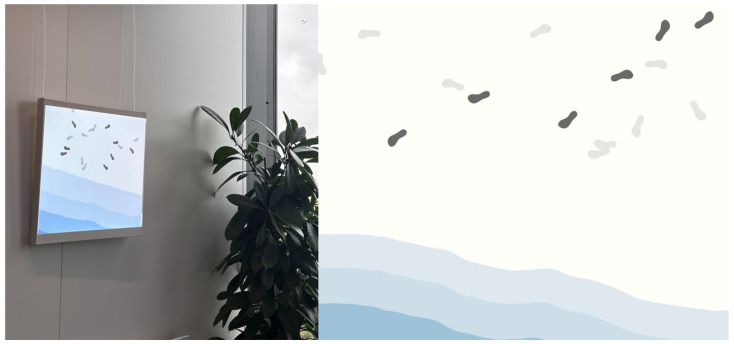
StepsConnect System. Digital art displayed in the young adult’s workspace visualizes the real-time step activity of their parent living apart, providing an ambient cue of ongoing presence.

To explore this possibility, we developed StepsConnect, an ambient system that integrates real-time step data into a digital artwork displayed within a young adult’s workspace, as shown in Figure 1. Parents contribute step data as part of the dyadic context, while young adults experience the visualization as an ambient presence cue. The present study focuses specifically on young adults’ perceptions within this unilateral exposure setting.

This leads to the research question guiding the present work:

How does ambient digital art visualization of step-based movement influence young adults’ sense of presence and perceived connectedness with their parents living apart?

This paper makes three main contributions:

1. We introduce StepsConnect, a real-time step-sensing-based ambient display system that translates everyday walking activity into expressive digital art, exploring how embodied activity signals can be represented as subtle presence cues in intergenerational contexts.
2. We report an exploratory user study with young adult–parent dyads that examines how ambient visualization of real-time step activity is perceived by young adults within everyday work environments, considering both quantitative patterns and qualitative accounts.
3. We provide preliminary design insights for human-centered sensing systems, clarifying how natural bodily activity signals and aesthetic representation may support momentary relational awareness and establishing a foundation for future investigations into bidirectional and long-term intergenerational communication systems.

## 2. Related Works

### 2.1. Step-Sensing-Based Ambient Awareness System for Remote Family Connectedness

Ambient awareness systems aim to support interpersonal relationships by enabling lightweight, peripheral awareness of others’ activities and presence [45,52]. Strong social ties with family members contribute positively to psychological well-being [53,54], and a variety of systems have explored how ambient intelligence can help maintain this sense of connection. For example, the Digital Family Portrait [55] provides family members with a qualitative sense of a senior adult’s daily activities, offering reassurance when living apart. Similarly, the CareNet Display [56] delivers contextual information such as activities, moods, and routines to members of an elder care network. These systems demonstrate that ambient communication can help individuals stay connected with family in a non-intrusive, continuous manner.

However, many early awareness systems relied on self-reporting or wizard-of-oz sensing, placing additional burden on users. For instance, both the Digital Family Portrait [55] and the CareNet Display [56] rely on wizard-of-oz sensing. The ASTRA awareness system [45] kept a diary of their informal social use of communication media. Users were asked to write down the time and describe what happened, who the other person was, and how they felt. To minimize effort, more recent research has increasingly employed quantified-self technologies to automate data collection and support ambient awareness with minimal user involvement, such as heart rate [57,58,59,60], breathing [61,62], activity and movement [26,51,63,64], presence [22], and environmental noise [65].

Among quantified-self signals, activity information is one of the most widely used cues for promoting connectedness. Davies et al. [19] demonstrated that sharing older adults’ daily activity levels through a bidirectional ambient display supports social awareness between caregivers and seniors. SoPresent [22] similarly used audio–video analysis to communicate presence and activity to family members of empty nesters. In particular, step count information has been shown to be effective for enhancing connectedness. Shinokawa et al. [66] found that sharing step information helped unfamiliar older men develop social bonds and reduce loneliness. Building on this finding, our work explores how step-sensing-based ambient awareness systems can integrate step data as embodied and emotionally meaningful presence signals to support connectedness between young adults and their parents living apart.

### 2.2. Representations of Digital Art in Ambient Displays

In ambient display systems, quantified-self data are most commonly presented using traditional visualizations, including numbers, time-series graphs, calendars, and textual summaries [67]. This reflects the dominant use of quantified-self data for self-tracking and reflection [68], and more recently as a resource for interpersonal awareness and social connection [34]. For example, SocialBike [69] displays real-time cycling data, and the Daily Activities Diarist [48] presents daily routines through textual diaries to support family awareness. While these approaches provide clear and interpretable information, they primarily support cognitive awareness and are less effective at conveying embodied or emotional experiences of presence. Also, they do not naturally integrate into everyday domestic environments.

To address these limitations, more recent work has explored abstract and object-based representations embedded in domestic artifacts, seeking to provide more ambient, emotionally resonant, and low-effort forms of awareness. For example, Animo [23] visualized emotional states using animated shapes derived from heart rate data, Eleuda [25] used a huggable cushion for affective communication, and United-Pulse [70] enabled partners to share heartbeats through wearable rings. Similarly, several ambient systems visualize activity through simple light patterns or color changes, such as representing activity levels via RGB light [71] or displaying social activity through ambient illumination [72]. These designs demonstrate that non-textual and ambient representations can support emotional engagement and peripheral awareness. However, their representational capacity is typically limited to low-dimensional cues (e.g., brightness, color, or motion), which constrains their ability to convey embodied movement, temporal rhythm, and emotional nuance.

In contrast, digital art offers a fundamentally different representational layer. Through rich dynamics, metaphorical forms, and aesthetic expressiveness [73], digital art can translate step-based bodily activity into subtle, emotionally meaningful presence signals that align with the embodied and spatial mechanisms underlying co-presence. Despite the growing interest in ambient awareness and activity-based connectedness, no prior work has examined how digital art can function as a presentation layer for everyday bodily activity cues in such systems.

Consequently, it remains unclear how activity-based presence signals can be rendered in artistic and metaphorical forms that enrich ambient awareness and support remote connectedness, particularly between young adults and their parents.

### 2.3. Summary and Research Gap

Prior work shows that step-based activity sensing combined with ambient displays can support low-effort awareness and connectedness between family members living apart. However, existing systems typically present such data through numbers, simple graphics, or light-based cues, which mainly support functional awareness and offer low-dimensional visual language constrains how richly everyday bodily activity can be experienced as a presence signal.

Digital art provides a richer representational layer for expressing everyday bodily activity as emotionally meaningful presence cues, yet research on translating such activity into artistic and aesthetic representations for connectedness enhancement remains sparse in existing quantified-self-based ambient connectedness systems.

This gap motivates our work in designing StepsConnect, a system that transforms real-time step data into dynamic digital artwork embedded in everyday environments to support presence, emotional warmth, and connectedness between young adults and their parents living apart.

## 3. Materials and Methods

### 3.1. StepsConnect System Design

#### 3.1.1. System Components and Functional Layers

**Figure 2:**
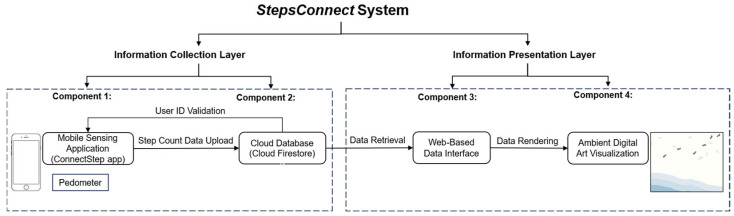
Four Components and Two Functional Layers of the StepsConnect System.

The StepsConnect system is designed as an end-to-end sensing and visualization pipeline that captures walking activity from a remote family member and presents it as an ambient digital artwork in real time, as shown in Figure 2.

These components are organized into two functional layers: (1) an information collection layer, consisting of the mobile app and cloud database; (2) an information presentation layer, consisting of the web-based interface and the digital art visualization, which together form an end-to-end sensing-to-visualization pipeline.

The information collection layer is responsible for detecting, recording, and synchronizing walking activity. The mobile app reads cumulative step counts from the device pedometer and transmits updates to Cloud Firestore, where each user’s data are stored in a dedicated document.

The information presentation layer subscribes to these cloud updates and renders them visually. The web interface retrieves the most recent step values and drives the digital art visualization, which presents the remote family member’s walking activity as an ambient, continuously updating artwork. In this way, physical movement in one location is converted into a subtle visual presence in another.

The StepsConnect system consists of four tightly coupled components that together support step data acquisition, transmission, processing, and visualization:

- Component 1: Mobile sensing application (StepsConnect app), which runs on the parent’s Android smartphone and captures step count data from the device’s built-in pedometer.
- Component 2: Cloud database (Cloud Firestore), which stores and synchronizes step count data across devices in real time.
- Component 3: Web-based data interface, which retrieves step data from the cloud and prepares it for visual visualization.
- Component 4: Ambient digital art visualization, which transforms step data into a continuously evolving visual scene shown on a wall-mounted screen.

#### 3.1.2. Step Count Data Processing Pipeline

**Figure 3:**
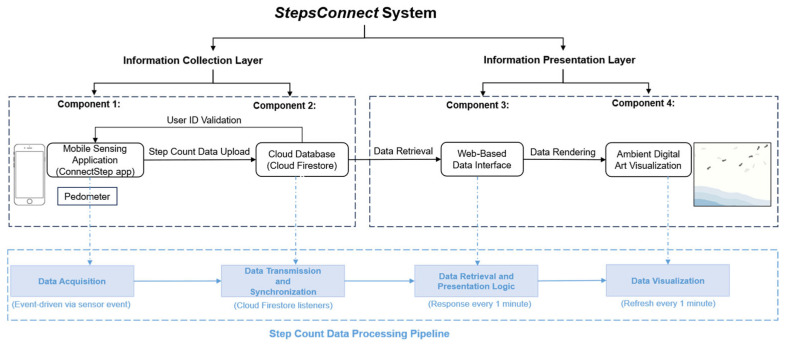
Step Count Data Processing Pipeline of the StepsConnect system. Blue elements denote step count data processing pipeline, and black elements represent core system components of the StepsConnect system.

After introducing the four system components and two functional layers, we next describe the end-to-end step data processing pipeline that connects the system components. The StepsConnect system implements an event-driven, end-to-end step data processing pipeline that links physical walking activity to ambient digital visualization, as shown in Figure 3.

The pipeline is composed of four sequential stages that ensure low-latency, reliable, and scalable synchronization between sensing, data transmission, and visualization.

First, data acquisition is performed on the parent’s Android smartphone using the built-in pedometer sensor, which continuously reports cumulative step counts whenever new steps are detected. These sensor updates are generated asynchronously by the operating system and do not rely on fixed-rate sampling.

Second, data transmission and synchronization are handled by the StepsConnect mobile application, which listens to pedometer events and writes each updated numeric step value to the steps field of the corresponding user document in a Cloud Firestore database. Cloud Firestore functions as a real-time, event-driven data store: whenever a value in the steps field is updated, it automatically triggers synchronization across subscribed clients rather than requiring explicit data requests. This mechanism provides persistent storage as well as low-latency propagation of step updates to the visualization layer.

Third, data retrieval and presentation logic are performed by the web-based client running on the ambient display. The client subscribes to the steps field of the corresponding user document via Firestore’s real-time listener interface. When new step values are pushed from the cloud, the web client receives them immediately and performs lightweight processing, including maintaining the cumulative step history and computing the most recent one-minute step increment, which are then passed to the visualization module.

Fourth, data visualization is performed on a wall-mounted peripheral digital art interface. Although step values are received in real time, the visualization logic updates the graphical representation at fixed one-minute intervals. This temporal aggregation highlights recent walking activity while maintaining a stable view of accumulated step history, enabling both short-term dynamics and long-term patterns to be perceived.

Together, these four stages form a modular sensing-to-visualization pipeline. This architecture enables near real-time reflection of walking activity on the ambient display with minimal latency and computational overhead. By separating sensing, data transmission, processing and visualization into modular stages, the pipeline ensures reliable synchronization between the parent’s physical movement and the child’s visual experience, supporting continuous and low-effort awareness across distance.

Section 3.1.3 and Section 3.1.4 detail the sensing and data transmission layers, and Section 3.1.5 and Section 3.1.6 describe the web-based and artistic visualization layers.

#### 3.1.3. Step Count Data Acquisition

**Figure 4:**
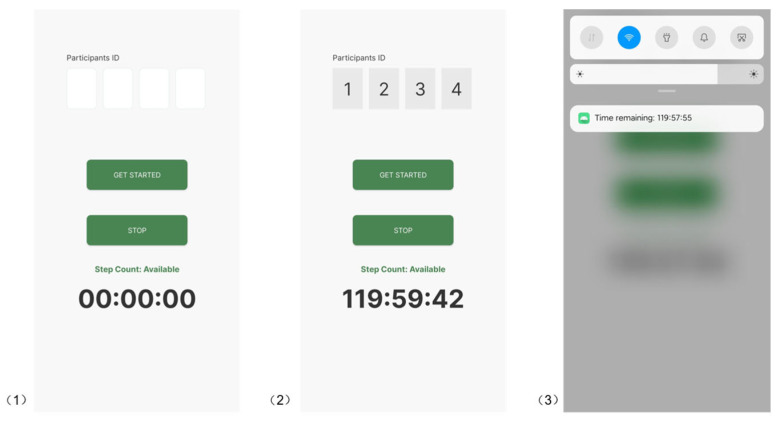
StepsConnect application operation: (**1**) StepsConnect launches and detects the pedometer sensor; (**2**) User enters the user ID and presses “GET STARTED” and the application begins working; (**3**) StepsConnect operates in the foreground.

The StepsConnect Android application, as shown in Figure 4, is developed using Kotlin within the Android Studio Integrated Development Environment [74]. The StepsConnect application leverages the device’s built-in pedometer to collect real-time walking activity data [75]. In this study, we define pedometer as the Android system’s step counter sensor (Sensor.TYPE_STEP_COUNTER). This is a system-level logical sensor provided by Android that reports the cumulative number of detected steps since the last device reboot through the sensor framework. Applications register a listener with Sensor Manager [76] to receive these step count values, and do not process raw motion sensor streams directly. Internal step detection logic is implemented by device firmware using built-in MEMS (Micro-Electro-Mechanical Systems) inertial sensors. Accelerometers measure linear acceleration along three axes, gyroscopes measure angular velocity, and magnetometers measure orientation relative to the Earth’s magnetic field. These signals are typically combined through vendor-specific sensor fusion algorithms to identify periodic gait patterns and infer steps. These processes are abstracted from applications and remain device-dependent. The pedometer interface thus provides a consistent, device-independent step count used in our data acquisition, without reliance on GPS or network connectivity.

Specifically, step count data are accessed through the Android Motion Sensor framework [75] by registering a sensor event listener for the system step counter sensor. This sensor provides cumulative numeric step count values representing the total number of steps detected by the device since the last system reboot. Unlike raw motion sensors (e.g., accelerometers), the pedometer does not operate at a fixed sampling rate. Instead, it functions in an event-driven manner, generating updates whenever new steps are detected by the operating system. In practice, this results in irregular but near-instantaneous updates during active walking periods, with no data interpolation or resampling applied by the application. Step detection and classification are performed internally by the Android operating system using proprietary algorithms that fuse signals from multiple motion sensors. StepsConnect does not apply any additional step detection logic, thresholding, or filtering; it solely reads the step count values provided by the system-level pedometer sensor.

The application runs continuously as a foreground service, allowing step data to be captured without requiring users to actively open or interact with the application. Upon installation, StepsConnect verifies the availability of a pedometer sensor on the user’s device. If the sensor is detected, the interface displays “Step Count: Available,” indicating that step data collection can proceed. For data processing, cumulative step count values are time-stamped and logged during system operation. Task-related walking activity is derived by computing the difference in cumulative step counts between the start and end of each task interval. These derived step counts form the basis for subsequent analysis and are stored locally before being transmitted to the backend server for further processing. The application is distributed as an Android Application Package (APK), enabling straightforward installation and consistent deployment across participants’ devices.

#### 3.1.4. Step Count Data Transmission and Cloud Synchronize

**Figure 5:**
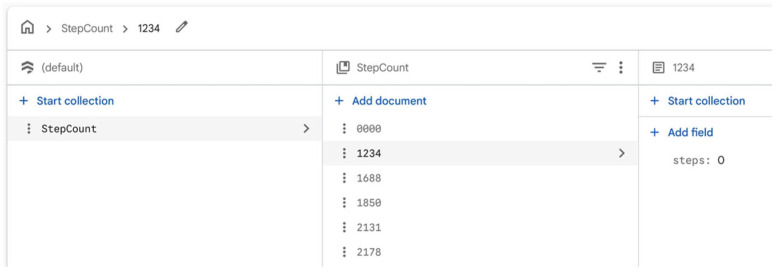
Screenshot of Cloud Firestore database structure.

To store and synchronize step count data, StepsConnect then integrates with Cloud Firestore from Firebase, a flexible and scalable NoSQL cloud database built on Google Cloud infrastructure to store and sync step count data (Google Developers, 2023) [77]. Within Cloud Firestore, we established a designated collection named “StepCount.” This collection organizes individual user data into distinct documents, each identified by a unique User ID. The user ID detection mechanism is integrated into the collection, serving as a validation mechanism. This method was chosen to ensure a convenient user experience. Before processing the step count input, StepsConnect checks if the provided User ID is stored in the Cloud Firestore database. When new step count data are obtained from the device pedometer, the updated numeric value is transmitted to a Cloud Firestore database and stored in the ‘steps’ field of the corresponding user document. The visualization module subscribes to this field via Firestore’s real-time listener interface. Whenever a new step value is received, the digital art display is automatically refreshed, allowing the parent’s walking activity to be reflected on the young adult’s screen with minimal latency, as shown in Figure 5.

#### 3.1.5. Web-Based Step Count Data Interface

**Figure 6:**
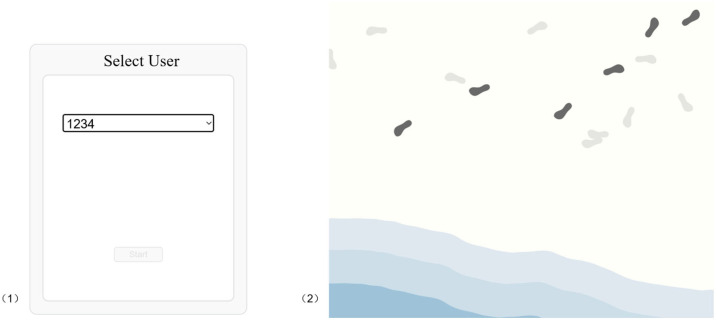
Screenshots of the web interfaces: (**1**) opening page (selected User ID ‘1234’), and (**2**) example digital art visual.

The system’s front end is a responsive web interface using HTML5 and JavaScript (ES6 standard). The web page generates the digital views by leveraging the real-time step count data stored in Cloud Firestore. Upon selecting the User ID, the interface will update the corresponding visuals every minute based on the latest step count data. As shown in Figure 6, the web interface provides both an entry page for selecting the User ID and a digital art view that reflects the associated walking activity.

#### 3.1.6. Ambient Digital Art Visualization of Step Count Data

To support ambient awareness of walking activity between family members living apart, the system visualizes real-time step data on a wall-mounted peripheral display as a continuously evolving digital artwork. The visualization design was guided by principles of perceptual saliency [78], embodied representation [79], and environmental integration within calm technology paradigms [80], ensuring that activity cues remain legible while blending naturally into everyday spaces.

Footprint shapes were selected as the primary visual element because step-count data are culturally and visually associated with footprints in widely used activity-tracking applications and health interfaces. This established graphic convention supports intuitive mapping between bodily movement and visual trace. Rather than introducing an abstract symbol, the footprint metaphor preserves the embodied meaning of walking as spatial presence.

To provide a coherent environmental context for these traces, footprints are embedded within a coastal scene. The seaside setting offers a spatially continuous surface where footprints are naturally expected, reinforcing semantic plausibility. In addition, coastal imagery is commonly associated with calmness and restorative affect, supporting the system’s goal of creating a gentle, ambient presence cue suitable for work or home environments.

To emphasize recent walking activity while preserving awareness of longer-term patterns, the visualization distinguishes between steps taken within the most recent one-minute interval and earlier accumulated steps. Steps recorded during the most recent minute are displayed as dark footprints, while earlier steps are rendered in light grey. This contrast-based encoding leverages established principles of visual saliency and figure–ground separation [81], allowing recent movement to stand out perceptually while preserving cumulative context.

To maintain visual clarity across varying activity levels, each footprint corresponds to 10 steps. This scaling ratio was determined based on reported distributions of daily step counts among middle-aged and older adults in prior epidemiological studies [82,83], which indicate substantial variability in daily walking volume. Direct one-to-one mapping between steps and footprints would result in excessive visual density during higher-activity periods. Aggregating steps in units of ten enables activity scaling while preserving perceptual clarity and aesthetic coherence over time.

**Figure 7:**
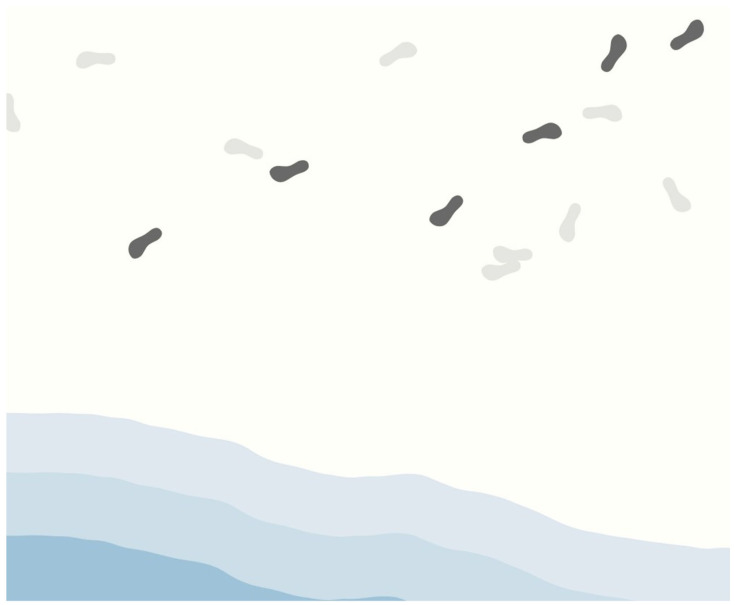
Dynamic digital art presentation of walking activity. Each footprint corresponds to 10 steps. Dark footprints represent the family member’s steps during the most recent one-minute interval, while light grey footprints indicate earlier accumulated walking history.

As shown in Figure 7, this layered and calibrated encoding allows users to perceive both moment-to-moment activity and cumulative walking history within a single, continuously updating ambient artwork.

### 3.2. Study Design

#### 3.2.1. Participants

This study was approved by the Eindhoven University of Technology Institutional Review Board (ERB approval number: ERB2023ID510) under the research protocol entitled “Perceived differences among raw data (number and text), graphic and digital art data presentations on the effects of connectedness between family members living apart.” The broader research protocol includes multiple representational formats of step-based sensing data, including numerical, graphical, and artistic presentations. The present manuscript focuses specifically on the digital art condition, examining how artistic visualization is perceived and experienced as an ambient presence cue. All procedures complied with institutional ethical guidelines for research involving human participants. Participants were informed about the study purpose, their rights, and data processing practices, and provided written informed consent prior to participation.

While StepsConnect was designed to support connectedness between family members living apart, in this study we tested with young adults who lived separately from their parents. Parents participated as data providers to create a realistic dyadic scenario but did not interact with the system directly.

We conducted an exploratory user study with 15 young adults (eight males and seven females), recruited from researcher’s network, students, and staff in the industrial design department and other departments at Eindhoven University of Technology. Age ranged from 20–31 years old. Nationalities varied from 8 countries, including China, Mexico, Netherlands, United Kingdom, Greece, Hungary, Croatia, and Thailand. Moreover, 13 lived in different countries from their family members, and the other two lived in other provinces. The duration of separation ranged from one week to one year.

#### 3.2.2. Experimental Setup and Procedure

1. Experimental Setup

**Figure 8:**
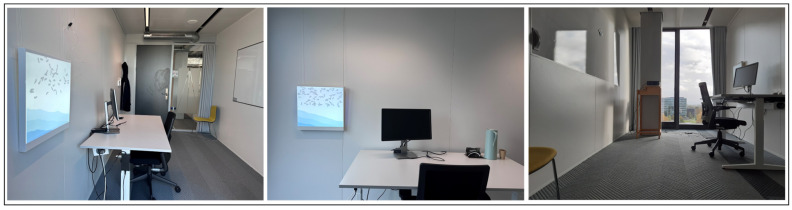
Experiment room and digital art is hanging in front of the working desk.

We arranged a simulated work environment laboratory located in the 7th-floor office room of the Atlas building at Eindhoven University of Technology, in which the dynamic digital art presentation was hung on the wall, as shown in Figure 8.

2. Procedure

The in-lab user test followed the procedure shown in Table 1. The experiment lasts for around 2 h with each participant. Participants must complete three steps: preparation, in-lab experience, and interview.

Three steps are shown as follows:

- First step: preparation

Before the in-lab user test, participants were asked to help their parents download the StepsConnect Android application and enable the necessary activity permissions on their parents’ Android phones. Participants’ parents also needed to learn how to enter the pre-determined user ID provided by the researcher and test whether the application functioned properly on their devices. After the installation and testing process, participants were asked to book three 2 h time slots with the researcher for the in-lab user experience. They were informed that during each 2 h session, they could arrange 1.5 h of self-directed laptop work. Participants were allowed to control their own work tasks but were instructed not to schedule meetings during the experiment.

- Second step: in-lab experience

Upon arrival at the laboratory, participants were first introduced to the research purpose and experimental procedure by the researcher to clarify the tasks they would complete during the study. Each participant was required to experience the StepsConnect system for 90 min during their simulated work session. Participants then read and signed the consent form. Afterward, they completed a questionnaire collecting general demographic information and their initial level of closeness with their parents living apart from them, measured using the Inclusion of Others Scale (IOS) [50]. Once the questionnaire was completed, participants were instructed to contact their parents by pressing the “Get Started” button on their parents’ phones to activate the app for offering their parents’ real-time steps information. After the researcher verified that the application was functioning correctly and had displayed the pre-determined dynamic presentation, participants began their 1.5 h work session in the office. Work time was structured using the Pomodoro Technique [84], consisting of 25 min focused work periods followed by 5 min breaks. After the work session, participants again completed the Inclusion of Others Scale (IOS) [85].

- Third step: interview

After completing the StepsConnect experience, participants took part in a semi-structured interview with the researcher. All interviews were audio-recorded for later analysis.

3. Walking data acquisition

During the study, walking data were generated by participants’ parents using their own Android smartphones with the StepsConnect application installed. Parents carried their phones during their normal daily activities and were not given any specific instructions regarding walking routes, speed, or duration, in order to preserve naturalistic behavior.

Step count data were continuously captured by the application while running in the foreground and transmitted in real time to the Cloud Firestore database. These data were then used to update the digital art visualization displayed to the young adult participants during the in-lab sessions.

#### 3.2.3. Measurements

We evaluated the effect of the StepsConnect system on young adults’ perceived connectedness using the Inclusion of Other Scale (IOS) [85] and gathered qualitative perspectives through semi-structured interviews.

- Inclusion of Other Scale (IOS)

**Figure 9:**
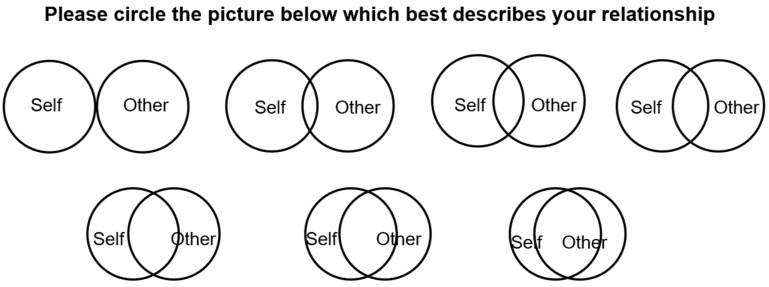
Redrawn diagram of the Inclusion of others in the Self (IOS) scale based on Aron et al. (1992) [85].

The IOS [85] is a widely used single-item pictorial measure designed to assess perceived connectedness. The scale presents seven diagrammatic pairs of overlapping circles labeled S (Self) and O (Parent), representing increasing degrees of self–other overlap. Participants select the diagram that best reflects their perceived relationship with the specified other person, with greater overlap indicating higher perceived closeness, as shown in Figure 9.

In this study, the IOS was used to assess young adults’ perceived connectedness to their parents living apart. Participants completed the IOS both before and after interacting with the StepsConnect system to examine potential shifts in perceived relational closeness following the ambient visualization experience.

- Semi-structured Interview

The interview questions aim to get users’ perspectives on (1) how participants experienced the StepsConnect system and (2) suggestions and expectations for the StepsConnect system. All interviews were audio-recorded for later analysis.

- Steps Counts of Young Adults’ Parents

In addition to subjective young adults’ perceived connectedness measures, parents’ walking activity was captured as an objective behavioral indicator of everyday presence. These data were used both to drive the real-time digital art visualization and to record parents’ physical activity during the 90 min experimental session.

For each parent, we extracted the total number of steps accumulated during the session and the mean steps per minute. These measures were used as objective references when interpreting young adults’ perceived connectedness.

#### 3.2.4. Quantitative Statistical Analysis

Changes in young adults’ perceived connectedness were analyzed using participants’ IOS scores measured before and after exposure to the StepsConnect system. Normality of the difference scores was assessed using the Shapiro–Wilk test.

Because IOS is an ordinal scale and the sample size was small, a Wilcoxon signed-rank test was used as the primary non-parametric test to compare pre- and post-intervention scores. For comparison with prior literature, a paired-samples *t*-test was also conducted.

Statistical significance was evaluated at α = 0.05. Effect sizes were reported as Cohen’s d for the *t*-test and r for the Wilcoxon test. All statistical analyses were performed using SPSS 23.0.

#### 3.2.5. Qualitative Data Analysis

All interview recordings were transcribed verbatim and analyzed using a reflexive thematic analysis approach following Braun and Clarke [86]. The first author conducted the coding. Transcripts were read repeatedly to achieve familiarization with the data, and initial codes were generated inductively through iterative review of participants’ descriptions of their experiences, emotions, and expectations. Codes were subsequently clustered into broader themes based on conceptual similarity and recurring patterns across transcripts. The coding process involved multiple analytic passes to refine theme boundaries and ensure coherence. Representative verbatim quotations are presented in the Results section to illustrate the identified themes.

#### 3.2.6. Steps Count Analysis

For each parent–child dyad, two step-based measures were computed from the logged pedometer data: (1) total step count over the 90 min session; (2) mean steps per minute, calculated by dividing the total steps by the duration of the session. These values were used to characterize the variability of parents’ physical activity across participants. Rather than modeling step counts as predictors of IOS change, the step data were used as objective behavioral references to contextualize the subjective young adults’ perceived connectedness outcomes reported by participants.

## 4. Results

This section presents the evaluation results based on the IOS scale and the semi-structured interviews.

### 4.1. IOS Scale

To examine changes in young adults’ perceived connectedness, the Inclusion of Others in the Self (IOS) scale [51] was administered before and after participants interacted with the StepsConnect system. IOS is a widely used single-item pictorial measure assessing perceived relational closeness.

Difference scores (post–pre) were calculated for each participant. A Shapiro–Wilk test indicated that the difference scores were not normally distributed (W = 0.667, *p* < 0.001). Accordingly, a non-parametric Wilcoxon signed-rank test was used as the primary inferential analysis.

The Wilcoxon test indicated a statistically reliable change in IOS scores (Z = −2.22, *p* = 0.026), with an effect size of r = 0.57. However, given the small sample size (*n* = 15) and the distribution of individual responses, this result should be interpreted cautiously.

**Figure 10:**
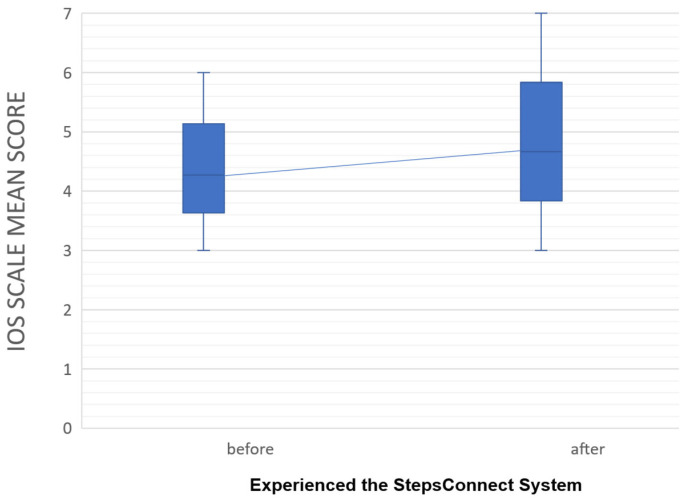
The mean score of the IOS Scale.

The mean IOS score increased from M = 4.27 (SD = 1.16) before interacting with the system to M = 4.67 (SD = 1.29) afterward, representing a modest shift at the group level. The mean IOS scores are illustrated in Figure 10.

**Figure 11:**
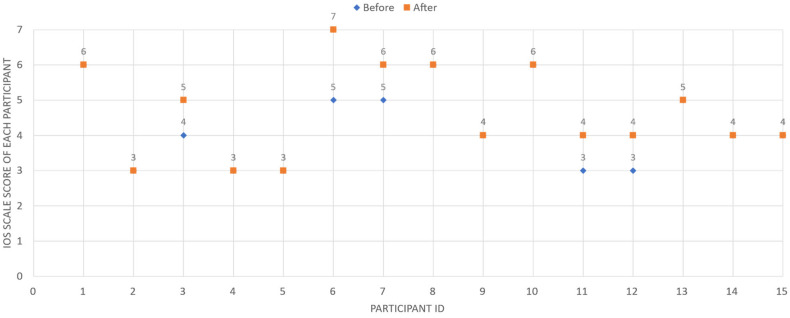
The IOS Scale score of each participant.

At the individual level, 5 out of 15 participants reported an increase in IOS score following the interaction, while the remaining 10 participants showed no change. No participants reported a decrease, as shown in Figure 11. These results indicate heterogeneous response patterns rather than uniform relational enhancement.

Taken together, the quantitative findings suggest that short-term exposure to step-based ambient awareness may influence perceived closeness for a subset of participants, while others maintained stable relational perceptions. The variability in responses highlights the exploratory nature of the study and the potential influence of baseline relational closeness and individual interpretive differences.

### 4.2. Semi-Structured Interview

The semi-structured interview results include participants’ perspectives on their experience with the StepsConnect system, as well as their expectations for future design improvements. Participants’ experiences clustered around four main dimensions: perceived connectedness and presence, system qualities, visual interpretation, and other emotional response. Participants’ expectations with the StepsConnect system are summarized in the following six aspects: desire for more types of information, multisensory, personalization, two-way interaction, more engagement, and improving accessibility for older adults.

#### 4.2.1. Experience of the StepsConnect System

1. Connectedness & Presence

- Reminder of Parents

Many participants reported that the ambient visualization frequently brought their parents to mind during working. Rather than requiring deliberate interaction, the display functioned as a passive yet persistent reminder of the parent’s existence and daily life. Participants described that the visualization naturally triggered thoughts about what their parents might be doing at that moment. For example, one participant noted, “It just makes me think about them” (P11), while another explained that the visualization encouraged them to imagine their parent’s current activities (P3, P4).

- Feeling of presence

Some participants interpreted the footprints and motion in the artwork as direct traces of their parents’ real-time bodily activity, which supported a sense of presence rather than abstract data awareness. The visualization enabled participants to perceive their parents as physically active somewhere else at the same moment. One participant described the footprints as resembling “my dad’s footprints” (P13), while others reported that the display allowed them to infer whether their parent was currently active and to imagine their ongoing daily activities (P3, P4).

- Feeling of connectedness

Some participants reported that the visualization led them to feel emotionally closer to their parents while viewing the display. These participants described the system as increasing their sense of involvement in their parents’ everyday lives, even without direct interaction. For instance, P3 noted that the system made them feel “more involved with my mom’s activities,” allowing them to infer what their parent was doing at that moment.

- Emotional distance

In contrast, some participants experienced the visualization as highlighting rather than reducing the distance to their parents. For these participants, the system evoked feelings of separation or longing rather than comfort. As one participant expressed, “It’s just a reminder of how far we are away from each other… I’ve just missed them” (P5).

2. System Qualities

- Decorative Integration

Some participants appreciated the idea of presenting real-time family information through a digital artwork that could be naturally placed in a work or living environment. They perceived the system not as a technical dashboard but as a decorative object that could coexist with everyday activities. For example, one participant noted that “the design is about putting some digital art in the office” (P3), while another described it as “a pretty nice idea” for having a subtle reminder of someone close while working (P15).

- Non-intrusiveness

Some participants emphasized that the system was non-intrusive and did not disrupt their routines. Instead of demanding attention or interaction, the visualization remained in the background, allowing awareness to emerge naturally. As P6 stated, it enabled connection “without disturbing the normal lives of both parties,” and P9 described that they “didn’t pay attention to it, but in a good way.”

- Low-pressure Interaction

For some participants, the ambient nature of the display reduced the social pressure typically associated with messaging or calls. They felt free to acknowledge their parents’ activity without being obliged to respond immediately. One participant explained, “You don’t feel the urgency to respond instantly… you can respond when you have time” (P15), while another described it as “a connection that is not mandatory and also fun” (P14).

- Surveillance concern

In contrast, some participants raised concerns about privacy and monitoring. One participant expressed that the system felt like “spying on someone, and digital art is trying to hide the feeling of spying” (P10).

3. Visual Interpretation

- Intuitive Interpretation

Several participants found the visual representation of step data easy to understand and transparent. The footprints and motion allowed them to quickly infer their parents’ level of activity without effortful decoding. For example, P15 described the visualization as “straightforward” and simple to interpret, while P4 noted that “the footprints make it transparent.”

- Imaginative Engagement

Some participants experienced the visualization as stimulating imagination about their parents’ ongoing activities. The human-like appearance of the footprints and their motion prompted participants to envision everyday scenarios such as walking, commuting, or shopping. As P14 emphasized, the visuals had a “very human-looking” quality, which helped participants imagine what their parents were doing at that moment (P3, P4, P12).

- Memory-evoking Qualities

For some participants, the artwork also triggered personal memories and associations related to their parents or hometown. For instance, P9 noted that the visualization reminded them of their mother’s love of swimming, while P7 described how the seaside scene evoked memories of playing at the beach with their parents.

4. Other Emotional Response

- Calm and Relaxation

Many participants reported that the visual design of StepsConnect, particularly the sea and wave elements, was calming and emotionally soothing. Many described the display as relaxing to watch and emotionally pleasant to have in their workspace. For example, P15 stated that “the moving sea makes me feel it is enjoyable to know the step count of my parent in this way,” while P1, P10, and P13 noted that the sea visuals helped reduce stress. Others similarly described the waves and motion as producing a calming atmosphere (e.g., P3, P9).

- Unsettling Emotions

However, some participants experienced some visual elements as uncomfortable or emotionally negative. In particular, the random placement of footprints was perceived by some as confusing or unsettling. For example, P2 and P14 reported disliking the randomness of the footprints, and P5 associated the scene with sadness and disorientation, describing it as resembling someone “lost on the beach.”

#### 4.2.2. Expectations for the StepsConnect System

1. Desire for additional information

Several participants expressed a desire to receive richer and more diverse information about their parents beyond step counts. In particular, participants mentioned interest in health- and safety-related data, such as heart rate or blood glucose levels. For example, P12 stated that seeing physiological information “could be interesting,” while P4 emphasized safety-related concerns. One participant (P3) explicitly mentioned chronic illness, noting that access to information such as blood glucose would be meaningful because “my dad has diabetes.” At the same time, some participants also expressed awareness of privacy boundaries (e.g., P15).

2. Desire for multi-sensory channel

Some participants suggested that adding additional sensory channels, such as sound, could strengthen the feeling of connection. For example, one participant proposed incorporating auditory cues like footsteps to reinforce the perception of their parent’s activity (P1).

3. Desire for more personalized visual elements

Several participants expressed a desire for more personalized visual representations. They suggested incorporating elements that reflect their parents, hometowns, or shared memories to increase emotional resonance. For instance, P8 proposed using familiar icons or scenes to enhance immersion, while P15 suggested that representations resembling the parents themselves (e.g., avatars) could further strengthen the sense of connection.

4. Desire for two-way interaction

Some participants emphasized the importance of two-way awareness. They felt that the system would foster stronger connectedness if parents could also receive feedback or messages from their children, allowing both sides to perceive each other’s presence. As P5 noted, one-way awareness limited the feeling of “being together,” while P15 imagined receiving unexpected messages from their parents through the system.

5. Desire for more engaging interactions

Several participants expressed interest in more engaging or interactive features. Beyond passive awareness, they wanted opportunities to actively share moments or perform activities together, such as exchanging photos (P15), exercising simultaneously (P2), or interacting through playful elements like games (P14). As P15 reported, “I think it will be a bit interesting if my parents could put the text there, like the text will appear like, oh how you do. It is about the Feeling of the unexpected.”

6. Improving accessibility for older adults.

Some participants reported practical barriers related to their parents’ ability to use the system, particularly difficulties installing the application on smartphones. As P7 noted, simplifying the installation and setup process would be critical for broader adoption among older adults.

### 4.3. Objective Step Activity Data Overview

To provide an objective reference for the subjective results, we report the walking data captured by the step-sensing system during the study period. These step counts reflect parents’ real-world physical activity during the 90 min session and are reported primarily as contextual information rather than as predictors of subjective outcomes.

We did not aim to statistically explain or predict changes in IOS based on walking behavior. Instead, the walking data are reported to provide contextual grounding for participants’ subjective experiences of presence, awareness, and perceived connectedness. As shown in Table 2, parents’ activity levels varied widely, ranging from very low activity (e.g., fewer than one step per minute) to highly active patterns exceeding 40 steps per minute. This wide variability ensured that the digital artwork exhibited substantially different degrees of motion and visual change across participants, thereby providing a meaningful objective backdrop against which subjective experiences can be interpreted.

**Figure 12:**
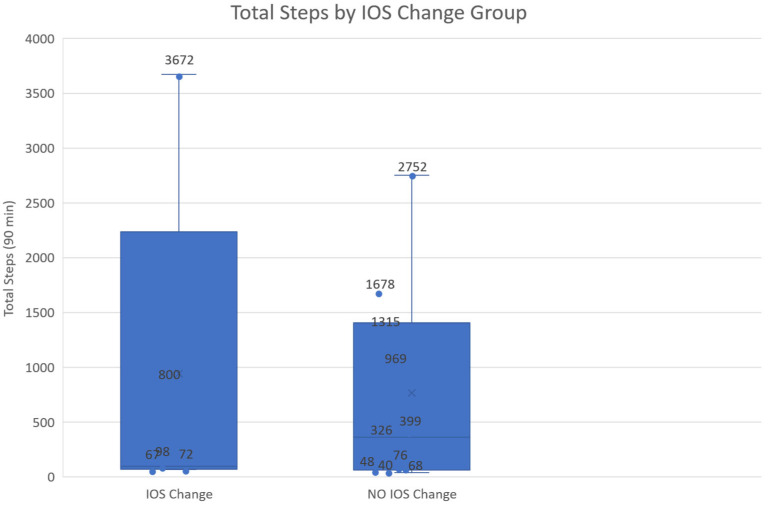
Distribution of total step counts during the 90 min session for participants with and without IOS change (IOS ≠ 0 vs. IOS = 0). Boxplots summarize group-level distributions, with individual data points overlaid to show participant-level variability.

While walking behavior was not treated as a primary explanatory variable, we conducted a supporting analysis to rule out the possibility that changes in young adults’ perceived social connectedness were simply driven by differences in the overall amount of physical activity, as indexed by total step counts during the session. Participants were divided into two groups based on whether their IOS score showed any change (IOS = 0 vs. IOS ≠ 0), and total step counts were compared between groups. An independent-samples Welch’s *t*-test revealed no significant difference in total steps between the two groups (*p* = 0.83). This result was confirmed by a non-parametric Mann–Whitney U test. No significant differences were found using either a Welch’s *t*-test or a Mann–Whitney U test. Visualization using boxplots with overlaid individual data points showed substantial overlap in step distributions between groups, particularly given the small and unequal sample sizes, as shown in Figure 12. Together, these results suggest that changes in young adults’ perceived social connectedness were not driven by the overall amount of physical activity.

### 4.4. Summary of Results

The mean IOS scores showed a modest shift following interaction with StepsConnect. At the individual level, five of the fifteen participants exhibited an increase in perceived closeness with their parents, while the remaining participants reported no change. This distribution reflects heterogeneous response patterns rather than a uniform change across the sample.

Parents’ walking activity recorded by the step-sensing system varied substantially across dyads during the 90 min session. These step counts are reported to provide an objective behavioral reference for participants’ experiences. Consistent with the supporting analysis reported above, no clear association was observed between the overall amount of walking activity, as indexed by total step counts, and changes in IOS scores.

Participants’ qualitative responses clustered into four main experiential dimensions: connectedness and presence, system qualities, visual interpretation, and other emotional responses. Participants described experiencing reminders of their parents and a sense of perceived presence through the step-based visualization. The system was characterized as decorative, non-intrusive, and low-pressure, while the visual cues were interpreted as intuitive, imaginative, and memory-evoking. At the same time, the visualization elicited both calming and unsettling emotional reactions, depending on individual participants and specific visual elements.

Participants also provided a range of suggestions and expectations for improving the StepsConnect system. Their feedback focused on expanding the types of information displayed, enhancing the sensory experience, incorporating more personalized visual elements, enabling more engaging interactions, and improving accessibility for older adults.

## 5. Discussion

This study investigated how real-time step-sensing data can be integrated into digital art to function as an ambient presence cue for connecting family members living apart. Building on prior work in ambient awareness and connectedness systems, our results show that StepsConnect effects at the level of relational awareness through low-effort, non-intrusive awareness and visually meaningful activity cues. Below, we discuss these findings of experience for the StepsConnect system and expectations for the StepsConnect system in light of prior research and our initial expectations. The limitations and future work are also presented in this section.

### 5.1. Discussion of Experience for the StepsConnect System

1. Connectedness & Presence

The present findings highlight an important distinction between situational relational awareness and stable relational closeness. While IOS scores showed modest and heterogeneous changes at the group level, many participants qualitatively reported heightened awareness of their parents and subtle experiences of presence during the session.

Several factors may account for this pattern. First, many participants reported already feeling close to their parents prior to the study, suggesting a potential ceiling effect that constrained measurable upward change within a short experimental timeframe. When baseline relational closeness is relatively high, observable variation in trait-like measures such as IOS may be limited. Importantly, these interpretations are grounded in a unilateral exposure context, in which only young adults received ambient activity cues. The present findings therefore reflect one-directional relational awareness rather than reciprocal relational change.

Second, short-term ambient exposure may first influence momentary relational awareness before altering more enduring perceptions of closeness. The IOS scale captures perceived relational closeness as a relatively stable interpersonal construct. Within the brief exposure period of the present study, shifts in perceived presence may not immediately translate into measurable changes in trait-like closeness.

Rather than indicating that the measurement was invalid, the divergence between quantitative and qualitative findings suggests that relational perception may operate across multiple layers. In this sample, ambient step-based visualization appears to function primarily at the level of momentary awareness during short interactions, whereas stable relational structure may require longer-term or reciprocal engagement to exhibit measurable change, particularly in already close parent–child relationships within this study.

This distinction between state-level awareness and trait-level relational perception refines the contribution of the present study by clarifying the level at which the system appears to operate. It also suggests that future evaluations of ambient sensing systems may benefit from incorporating both state-sensitive and trait-oriented measures to capture multi-layered relational effects.

This study found that StepsConnect often acted as a natural trigger for communication between young adults and their distant parents. Participants (P1, P8) frequently reached out during the experiment, either to verify whether the system was functioning or to ask about noticeable changes in movement. In this study, subtle visual cues can prompt spontaneous contact and extend everyday conversations.

Although parents’ step counts varied widely, changes in IOS were not simply proportional to the overall amount of activity. This suggests that perceived presence may depend less on activity magnitude and more on temporal qualities such as variability and rhythm. Future research should examine longer-term deployments and explore reciprocal cues to assess whether these reminder-based micro-moments accumulate into more sustained forms of parent–child connectedness.

2. System Qualities

A core design goal of StepsConnect was to embed family awareness into everyday environments in a way that is visually acceptable, emotionally gentle, and behaviorally lightweight [87,88]. Consistent with this goal, participants described the system as decorative, non-intrusive, and low-pressure. The digital artwork was perceived as something that could naturally exist in a workspace or home environment rather than as a technical monitoring interface, which reduced the psychological barrier to keeping it visible for long periods. This aesthetic framing allowed step-based information to be perceived as part of the environment rather than as a demand for attention. Participants also emphasized the low-pressure nature of the system. Unlike messaging or calling tools that imply social obligation and reciprocity [23,25,70], StepsConnect did not require users to respond, reply, or synchronize with their parents. Instead, the visualization provided a continuous but optional sense of awareness, enabling participants to remain connected without experiencing communication pressure or emotional burden. This design aligns with prior work on calm technology [80,89] and peripheral awareness [78,90], which highlights the value of background information that supports social presence without interrupting primary activities.

At the same time, a small number of participants expressed concerns about surveillance, indicating that even low-resolution sensing such as step counts can be interpreted as monitoring. It is important to note that privacy protection was considered from the outset of the system design. The system adopts a data-minimalist approach: only aggregated step-count values are transmitted, without location tracking, detailed temporal logs, or raw motion sensor streams. The visualization represents cumulative symbolic activity rather than reconstructing movement trajectories, thereby limiting the granularity of shared information. Nevertheless, the emergence of perceived surveillance concerns suggests that privacy perception is shaped not only by the technical properties of the data but also by relational context, expectations, and individual sensitivity. This highlights an important design tension: while ambient sensing can reduce communication effort, it may still evoke monitoring-related interpretations. Future work may therefore explore additional mechanisms to support user agency and trust, such as adjustable sharing thresholds, explicit opt-in controls, and clearer transparency cues indicating what information is being shared.

3. Visual Interpretation

Participants’ visual interpretations of the StepsConnect display illustrate how step-based sensing was experienced as a cue of presence within this study. The intuitive readability of the footprint and motion cues appeared to enable participants to grasp their parents’ activity levels with minimal cognitive effort, supporting low-load, peripheral awareness rather than focused data inspection. Participants in this study generally described the visualization not as numerical information, but as an immediately legible signal of “someone moving,” suggesting that the visual encoding functioned as an awareness channel rather than as a conventional data display.

Beyond this functional layer, participants frequently engaged in imaginative and memory-based interpretations of the artwork. The footprints and seaside scene were experienced not only as indicators of movement, but also as traces of the parent’s presence, prompting users to imagine what their parents were doing and to recall personal memories associated with them. These responses suggest that the artistic representation may extend step data beyond activity reporting toward emotionally resonant interpretation.

Motion emerged as an important aesthetic parameter. Variations in movement speed and density were interpreted as indicators of liveliness or inactivity, allowing participants to perceive rhythms of daily life rather than absolute quantities. This observation aligns with prior work on informative art [90], which highlights how dynamic and abstract visual elements can communicate real-time activity in ways that are both perceptually interpretable and affectively engaging.

Prior ambient awareness systems have employed textual [48,69], graphical [48,69], or light-based representations [71,72] to communicate activity information. Rather than positioning digital art as superior to these approaches, the present findings suggest that artistic visualization constitutes a distinct representational modality. Within this exploratory study, digital art appeared to support an integration of functional awareness and personal meaning. Systematic comparison across representation formats remains an important direction for future investigation.

At the same time, it is important to recognize that the present visualization represents an initial exploratory design iteration rather than a fully optimized visual encoding strategy. While many participants interpreted the footprints and motion cues positively, a small number experienced the random spatial placement of footprints as unsettling or emotionally ambiguous. These reactions do not simply indicate design failure; instead, they reveal the sensitivity of ambient visualization to spatial rhythm, predictability, and affective tone.

The presence of both calming and unsettling responses suggests that subtle variations in spatial arrangement and movement dynamics can significantly influence emotional interpretation. This observation contributes to the design understanding of sensing-based ambient systems by highlighting the need to carefully calibrate metaphor selection, spatial structure, and mapping granularity. Future research may systematically examine alternative visual metaphors, structured versus stochastic spatial distributions, and different step-to-visual aggregation strategies to better understand how design parameters shape perceptual and emotional outcomes.

4. Other Experience Emotions

In addition to effecting at the level of relational awareness, the visualization also evoked a broader range of emotional responses. Many participants described the sea and wave elements as calming and pleasant, which aligns with prior work [91,92] showing that aesthetic, ambient displays can regulate emotions and create a gentle sense of social presence. These soothing qualities helped participants in this study remain open to interpreting the visualization as a subtle reminder of their parents. However, some elements were perceived as confusing or unsettling, particularly the randomly placed footprints. This suggests that overly abstract or unpredictable visuals can hinder interpretability and weaken emotional resonance. The results extend existing research by demonstrating that emotional tone and aesthetic coherence are crucial for using digital art as a non-intrusive channel for influencing relational awareness. When visuals are stable and metaphorically clear, they can support everyday comfortable emotions; when they are ambiguous, they risk disrupting the intended emotional effect.

### 5.2. Design Implications

Based on our findings, we identify several design implications for future intergenerational ambient systems aimed at supporting engagement and relational awareness between young adults and their parents living apart.

1. Provide richer activity information through multi-sensory and personalized feedback. Participants valued access to additional contextual details about their parents’ daily activity and well-being. Ambient systems should present activity cues using multiple sensory channels, such as subtle sound, light, animation, or haptics. This includes enabling personalization of visual elements that reflect family identity, memories, or cultural familiarity. Designers should therefore support configurable levels of detail that allow families to balance awareness with privacy.
2. Support reciprocal, two-way awareness to foster mutual presence. Designers should incorporate bidirectional awareness and lightweight responses that allow both parties to acknowledge one another, creating a sense of shared presence rather than passive observation.
3. Create opportunities for deeper, voluntary engagement layered on top of ambient cues. Ambient cues should operate effortlessly in the background, while richer interactions should be readily available when users want to transition into more meaningful, focused moments. This balance addresses the tension between low-effort expectations and the desire for emotionally engaging experiences.
4. Intergenerational systems should prioritize accessibility, including simplified onboarding, installation-free interfaces, pre-configured devices, or alternative channels that minimize cognitive load for older adults. Lowering these barriers is essential for adoption and sustained use.

Collectively, these design implications emphasize that intergenerational ambient systems should be subtle yet expressive, informative yet privacy-aware, personalized yet low-effort, and accessible across generations. By balancing ambient awareness with optional deeper interactions, designers can better support warmth, familiarity, and relational awareness in everyday remote family communication.

### 5.3. Limitations

This study has several limitations. Firstly, the sample size was small (*n* = 15) and restricted to a specific age group, which limits the generalizability of the findings. In addition, participants and their families were highly educated, reflecting a convenience sampling strategy. This demographic profile may have influenced their familiarity with digital technologies and should be considered when interpreting the results. The high level of technological fluency among participants may have positively influenced their acceptance and interpretation of ambient sensing systems. Therefore, the findings should be interpreted as context-specific and may not generalize to populations with lower technological familiarity.

Secondly, a further limitation concerns measurement sensitivity. Although the IOS scale aligns conceptually with perceived relational closeness, its trait-oriented nature and the relatively high baseline closeness reported by many participants may have constrained observable short-term variation. Future studies may incorporate additional state-sensitive measures to more precisely capture momentary relational awareness within brief ambient interventions.

Thirdly, both IOS scores and interview data rely on self-report, which may introduce biases such as socially desirable responding. Although walking data were logged as an objective behavioral reference, future research may benefit from incorporating additional behavioral or physiological indicators to triangulate relational experience.

Fourthly, the study was conducted as a 90 min laboratory-based session in a simulated work environment. This short-term exposure does not allow conclusions regarding long-term engagement, habituation effects, or sustained relational influence. Extended in-home deployment over days or weeks would be necessary to examine sustained engagement, habituation processes, and evolving relational meaning.

Finally, a further limitation concerns the qualitative analytic approach. The interview data were analyzed using a reflexive thematic analysis conducted by a single researcher without formal inter-rater reliability testing. While this approach aligns with interpretive qualitative traditions, it may increase the potential for researcher bias. Additionally, given the modest sample size and exploratory design, the identified themes should be understood as context-specific interpretive patterns rather than indicators of thematic prevalence or generalizable relational mechanisms. Future studies may incorporate collaborative coding processes or more diverse samples to further strengthen analytic robustness.

### 5.4. Future Work

The present study focuses specifically on understanding the perceptual characteristics of digital art-based visualization as an initial exploratory step within a broader research trajectory. Building on this investigation, future research will extend the work in several directions.

Firstly, future studies will systematically examine representational differences across formats (e.g., numerical, graphical, and artistic visualizations) to investigate how distinct presentation strategies shape interpretation, emotional engagement, and relational awareness.

Secondly, future research will investigate visual encoding mechanisms within artistic representations. This includes examining alternative visual metaphors, structured versus stochastic spatial distributions, and different step-to-visual aggregation strategies. Comparative analysis of more direct embodied mappings (e.g., step-to-footprint traces) and more indirect environmental transformations (e.g., step-driven changes in waves or landscape elements) may help clarify how varying levels of abstraction influence perceptual clarity and emotional interpretation.

Thirdly, subsequent work should focus on older adult populations and long-term in-home deployment. The present study was conducted with young adults in a controlled laboratory setting, while parents participated only as data providers. Future studies should examine how older adults experience and interpret ambient sensing systems when deployed in domestic environments over extended periods. Longitudinal in-home studies will be important for evaluating sustained engagement, usability, technological accessibility, and the evolving relational meaning of ambient presence cues within everyday family life.

Finally, system development will incorporate bidirectional interaction to examine how reciprocal awareness influences relational perception and shared presence experiences over longer-term, real-world deployment.

## 6. Conclusions

This study explored how real-time step-sensing data, when embedded into ambient digital art, can be integrated into everyday environments to support subtle forms of relational awareness between family members living apart. We presented StepsConnect, a sensing-based ambient display system that transforms parents’ step data into dynamic visual representations, enabling young adults in the present sample to perceive traces of everyday activity as a low-effort presence signal within a workspace context.

Findings from a 90 min laboratory study with 15 parent–child dyads indicated heterogeneous responses at the quantitative level, with modest group-level shifts in perceived closeness and substantial variability across individuals. Qualitative results suggested that, within this technologically fluent participant group, step-based visualizations functioned primarily as ambient reminders and cues of presence, supporting momentary awareness rather than immediate transformation of stable relational closeness. Overall, the study serves as an exploratory step in understanding how ambient step-based signals may be experienced as cues of presence under controlled laboratory conditions.

Rather than demonstrating definitive relational change, the study provides preliminary insight into how raw sensing signals can be translated into emotionally interpretable ambient cues. The results suggest that step-based activity visualization may operate initially at the level of situational awareness, potentially laying groundwork for longer-term relational influence under extended or reciprocal deployment conditions.

This work contributes to human-centered sensing research in three ways. First, it empirically examines how low-demand ambient representations of sensing data are perceived within a specific work-context setting. Second, it identifies system-level and visual encoding considerations that shape users’ interpretations of activity-based presence signals. Third, it clarifies the conceptual distinction between momentary relational awareness and more enduring perceptions of closeness, offering guidance for future measurement and system design.

Importantly, this study represents a focused component within a broader research trajectory. The present investigation examines unilateral ambient awareness mechanisms within a relatively small and technologically familiar participant group, serving as a foundational step toward future development of bidirectional systems, comparative visualization studies, and longer-term in-home deployment. Subsequent research will examine reciprocal interaction, multi-modal feedback, and sustained engagement across more diverse relational and demographic contexts.

Taken together, these results suggest that, within this context, aesthetic sensing-based ambient systems may function as a complementary layer to existing communication technologies within comparable contexts. Rather than replacing direct communication, such systems may provide subtle and peripheral cues of presence that coexist alongside established interaction channels. Further investigation in more diverse real-world settings is needed to assess long-term and cross-contextual relevance

## Figures and Tables

**Table 1:** Experiment Procedure.

Experiment Procedure			Duration
Preparation	App installation on parent’s phone and usage teach		Flexible
In-lab Experience	Consent form sign + general information fill + Initial IOS scale fill		10 min
	Check the APP on the parent’s phone works		Flexible
	Experience presentation	Work (25 min) + Rest (5 min)	90 min
		Work (25 min) + Rest (5 min)	
		Work (25 min) + Rest (5 min)	
	IOS Scale fill after experience		5 min
Interview	Semi-structured Interview (audio record)		20 min

**Table 2:** Parent Walking Activity and Young Adults’ Perceived Connectedness Change (IOS).

Parent ID	Total Steps/90 min	Mean Steps/min	Young Adults’ IOS
P1	48	0.53	0
P2	326	3.62	0
P3	67	0.74	+1
P4	399	4.43	0
P5	1315	14.61	0
P6	800	8.89	+2
P7	72	0.80	+1
P8	76	0.84	0
P9	1678	18.64	0
P10	68	0.76	0
P11	98	1.09	+1
P12	3672	40.80	+1
P13	40	0.44	0
P14	969	10.77	0
P15	2752	30.58	0

## Data Availability

The data generated and analyzed during this study are not publicly available due to ethical and privacy restrictions, as they contain personal and potentially identifiable information from family members, including interview recordings and questionnaire responses. Anonymized and aggregated data supporting the findings of this study are available from the corresponding author upon reasonable request and with approval from the Eindhoven University of Technology Institutional Review Board (ERB2023ID510).
